# Metabolomics, a New Promising Technology for Toxicological Research

**DOI:** 10.5487/TR.2009.25.2.059

**Published:** 2009-06-01

**Authors:** Kyu-Bong Kim, Byung Mu Lee

**Affiliations:** 11 National Institute of Toxicological Research, Korea Food and Drug Administration, Seoul, 122-704 Korea; 21 Division of Toxicology, College of Pharmacy, Sungkyunkwan University, Chunchun-dong 300, Changan-ku, Suwon, Gyeonggi-do, 440-746 Korea

**Keywords:** Metabolomics, Biomarker, Toxicology, High-throughput screening (HTS), Risk assessment

## Abstract

Metabolomics which deals with the biological metabolite profile produced in the body and its relation to disease state is a relatively recent research area for drug discovery and biological sciences including toxicology and pharmacology. Metabolomics, based on analytical method and multivariate analysis, has been considered a promising technology because of its advantage over other toxicogenomic and toxicoproteomic approaches. The application of metabolomics includes the development of biomarkers associated with the pathogenesis of various diseases, alternative toxicity tests, high-throughput screening (HTS), and risk assessment, allowing the simultaneous acquisition of multiple biochemical parameters in biological samples. The metabolic profile of urine, in particular, often shows changes in response to exposure to xenobiotics or disease-induced stress, because of the biological system’s attempt to maintain homeostasis. In this review, we focus on the most recent advances and applications of metabolomics in toxicological research.

## INTRODUCTION

Metabolomics (or metabonomics), a newly categorized '-omis', is derived from the Greek roots 'meta' meaning change and 'nomos' meaning rules or laws, to provide insight into the generation of pattern recognition-based models that are able to classify or predict changes in biological metabolism (Nocholson *et al.*, 1999; Watkins and German, 2002; Lindon *et al.*, 2004). Metabolomics is generally defined as the systemic investigation of the unique metabolite network or fingerprint which explains specific biological or etiological status with change of metabolome, the collection of all metabolites produced in biological systems (Lindon *et al.*, 2003). Metabolomes, relatively small molecular weight proteins of 100-1000 and the end products of biological organism's gene expression, are generally analyzed by mass spectrometry. Nicholson *et al.* (1999) defined 'metabonomics' as the quantitative measurement of the dynamic multi-parametric metabolic response of living systems to patho-physiological stimuli or genetic modification. There is no significant difference between the terms metabolomics and metabonomics, but the term metabolomics rather than metabonomics has been used because 'metabolomics' is more commonly used. Metabolomic strategies aim to detect changes in the distribution and concentration of a broad range of endogenous metabolites and can be applied to multiple levels of biological system: from single cell to whole organism (Celia *et al.*, 2002). The 'omics' suffix has come to signify the determination of a targeted level of biological molecules and information (Yang *et al.*, 2009). Therefore, genomics measures the entire genetic makeup of an organism, while proteomics analyzes all the proteins expressed under given conditions (Hrmova and Fincher, 2009; Rampitsch *et al.*, 2009). Metabolomics is not exceptional. Its relationship to the other '-omics' (toxicogenomics and toxicoproteomics) is represented in Fig. 1. Metabolism is the biochemical modification of chemical compounds by interaction with catalytic enzymes in living organisms or cells. This includes the biosynthesis of complex organic molecules (anabolism) and their breakdown (catabolism) into small ones. Metabolism usually consists of sequences of enzymatic steps, also called metabolic pathways. The total metabolisms are all biochemical processes of an organism,

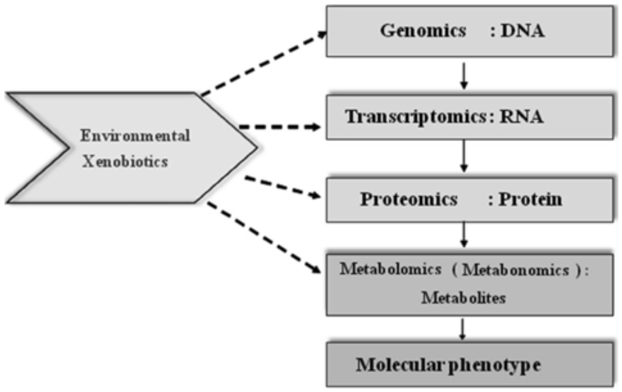

**Fig. 1.** The "OMICS" technologies involved in toxicological research. The integration of "omics" sciences might lead to a better comprehensive understanding of toxicological sciences.

whereas the cellular metabolism includes all chemical processes in a cell. Important metabolic pathways are composed of general pathways (carbohydrate, fatty acid, and citric acid metabolisms), catabolism, anabolism, and drug metabolism (Nealson and Conrad, 1999; Raman *et al.*, 2005; Testa and Kramer, 2006). A metabolome is generally termed as the whole set of metabolic small entities in a cell, tissue, organ, organisms, and species (Allen *et al.*, 2003). It includes small circuits of pathway networks. The very large portion of metabolome study has been metabolic engineering to produce industrially meaningful compounds (Wu *et al.*, 2005). Metabolomics is the method of studying, profiling and fingerprinting metabolites in various physiologic states (Fiehn *et al.*, 2002) (Table 1). Metabolite profiling is a main tool for the analysis of a class of metabolites. Metabolomics aims to include all classes of endogenous metabolites and utilizes metabolic fingerprinting of them to maintain a rapid classification of biological samples according to their origin and biochemical relevance (Nicholson *et al.*, 1999; Lindon *et al.*, 204). In order to optimize and utilize metabolomics, a stable and reproducible metabolite fingerprint must be established (Bino *et al.*, 2004). Metabolomic techniques are looking for a way to analyze changes to those endogenous biomolecules caused by xenobiotic toxicity or drug efficacy. In toxicological research, metabolomics is also viewed as holding great promise, including use in specific biomarker discovery for clinical diagnostics and drug discovery. In this review, the brief analytical technologies for metabolomics are summarized, the current toxicological applications of metabolomics are described, and then the prospective future of metabolomics for toxicology is discussed.

**Table 1.** General terminology for metabolite analysis

| General areas | Description |
|---|---|
| Target compound analysis | - The quantification of specific metabolites |
| Metabolic profiling | - Quantitative or qualitative determination of a group of related compounds or of members of specific metabolic pathways |
| Metabolomics | - Qualitative and quantitative analysis of all metabolites |
| Metabolic fingerprinting | - Sample classification by rapid and global analysis without extensive compound identification |

**Table 2.** Analytical methods used for characterization of metabolites

| Analytical methods | Feature | Ref. |
|---|---|---|
| NMR spectroscopy | - Cheap after initial purchase<br>- Robust and reliable<br>- Minimal sample preparation<br>- High throughput<br>- Significant metabolite overlap<br>- Large initial outlay | Reo *et al.*, 2002<br>Nicholson *et al.*, 1989<br>Raamsdonk *et al.*, 2001 |
| GC-MS | - Excellent sensitivity<br>- No need to derivatise<br>- More global than NMR or GC-MS<br>- Either specific or global<br>- LC reproducibility is less than GC<br>- Ion suppression can impede some metabolite detection | Fiehn *et al.*, 2002<br>Fiehn *et al.*, 2000 |
| LC-MS | - Good sensitity<br>- Cheap to purchase<br>- Good identification software<br>- Good chromatograms compared to LC-MS | Wilson *et al.*, 2003 |

## ANALYTICAL TECHNOLOGIES

Metabolomics is a multi-disciplinary technology, requiring cooperation between toxicologists, pharmacologists, chemists, biologists and informaticians. Current metabolomics practice has mainly relied on mass spectrometry (MS) and nuclear magnetic resonance (NMR) spectroscopy. The MS requires a preseparational procedure of metabolites using gas chromatography (GC) or liquid chromatography (LC) (Table 2). It's almost impossible to detect the whole population of metabolites in a system with single analytical method. Isolation of metabolites from biological samples requires the preparation of an extract. The choice of solvent used for this initial extraction process directly affects the chemical classes of compounds present in that extract. Furthermore, no spectroscopic method is available for the detection of all classes of metabolites.

Therefore, a variety of comprehensive and targeted methods need to be applied and the data integrated in order to provide a complete profile of metabolic status. A schematic representation of the sample preparation process for metabolite analysis, from tissue treatment to data analysis, is given in Fig. 2. In any case, comprehensive quantitative and qualitative determination of metabolites in biological samples may require parallel implementation of several processing and detection methods (Dunn and Ellis, 2005). All metabolomics studies produce complex multivariate data sets that need to be interpreted using chemometric and bioinformatic methods (Nicholson *et al.*, 1999).

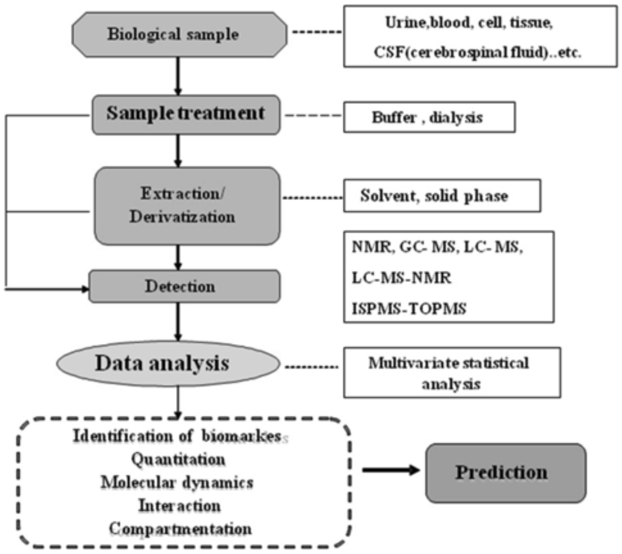

**Fig. 2.** Schematic diagram of the procedure for metabolomic approach.

***NMR.*** Proton ($^1H$) NMR can detect any metabolites containing hydrogen (Table 3). Signals can be assigned by comparison with libraries of reference compounds, or by two-dimensional NMR. The $^1H$ NMR spectra of crude biological tissue extracts are inevitably crowded with many overlapped signals, not only because there is a large number of contributing compounds, but also because of the low overall chemical shift dispersion. $^1H$ NMR spectra are also complicated by spin-spin couplings which add to signal multiplicity, although they are an important source of structural information (Griffin *et al.*, 2003). In $^{13}C$ NMR, the chemical shift dispersion is twenty times greater and spin-spin interactions are removed by decoupling. Despite these advantages, the low sensitivity of $^{13}C$ NMR prevents its routine use with complex extracts (Kenney *et al.*, 2003; Bundy *et al.*, 2003).

***Gas chromatography.*** Gas chromatography (GC) provides compound separations with high-resolution and can be used in conjunction with a flame ionization detector (GC/FID) or a mass spectrometer (GC/MS). Both detection methods are highly sensitive and able to detect almost any organic compound, regardless of its class or structure. However, some of the metabolites found in biological samples are too involatile to be anal-

**Table 3.** NMR-based metabolomics and potential biomarkers

| Type | Sample | Biomarkers | Ref. |
|---|---|---|---|
| Neurochemicals TTX(tetrodotoxin): rat frontal cortex. | Cerebrospinal fluid (CSF) | - Glutamate, isoleucine, valine, alanine, α- and β-hydroxybutyrate↓ | Khandelwal *et al.*, 2004 |
| Myocardial ischemia. | Tissues | - Plasma: lactate, acetate, acetone<br>- Brain: glycerol, succinate, propionate, lactate | Price *et al.*, 2005 |
| Dominant-submissive relationships: rats | Urine | - Milk sugar consumption, urinary galactose normalized to creatinine | Leo *et al.*, 2005 |
| Dietary influence (healthy British, Swedish subjects) | Urine | - Trimethylamine-N-oxide (TMAO)↑ taurine↑ | Lenz *et al.*, 2004 |

ysed directly by GC methods. Therefore, the compounds have to be converted to less polar, more volatile derivatives before they are applied to the GC column.

***High performance liquid chromatography (HPLC).*** HPLC, with UV detection, is a common method used for targeted analysis of biological samples and for metabolic profiling of individual classes (Fan *et al.*, 2005). Derivatisation is not essential. Selection of compounds arises initially from the type of solvent used for extraction and then from the type of column and detector. For example, HPLC/UV will only detect compounds with a suitable chromophore; a column selected for its ability to separate one class of compounds will not generally be useful for other types. HPLC profiling methods all rely, to a great extent, on comparisons with reference compounds. The full UV spectrum (measured for each peak when UV-diode array detectors are used) gives some useful information on the nature of compounds in complex profiles, but often indicates the class of the compound rather than its exact identity (Plumb *et al.*, 2002).

***LC/MS, LC/MS/MS and LC/NMR.*** LC/MS, LC/MS/MS and LC/NMR are powerful solutions to the problems of detector generality and structure determination. LC/MS can be used to detect compounds that are not well characterized by other methods (those that are not easily derivatised), lie above the available GC/MS mass range, or do not contain good chromophores for conventional HPLC (Wilson *et al.*, 2005). The electrospray ionization (ESI) technique has made polar molecules accessible to direct analysis by MS, as well as being compatible with HPLC separations (Buchholz *et al.*, 2001). Quantification of multiple compounds in crude extracts can, in principle, be achieved the same way as GC/MS, with high sensitivity. However, automation of the procedure presents greater practical difficulties (Jonsson *et al.*, 2004). LC/MS/MS provides additional structural information that can be a very useful aid in the identification of new or unusual metabolites, or in the characterization of known metabolites in cases where ambiguity exists. LC/NMR combines the superior structure-determining power of NMR with HPLC in a flow system (Exarchou, 2005).

***Direct injection into high-resolution MS.*** It is possible to obtain metabolite ‘mass profiles’ without any chromatographic separation. Such profiles are obtained by injecting crude extracts into the source of a high-resolution mass spectrometer (Dunn and Ellis, 2005). Electrospray ionization (ESI) or atmospheric pressure chemical ionization (APCI) generates mainly protonated, deprotonated or adduct molecules, such as $[M+H]^+$, $[M+cation]^+$ or $[M-H]^-$ for each species present in the mixture, with little or no fragmentation (Nordstrom *et al.*, 2008). Thus, a fingerprint spectrum is obtained with a single or a few peaks for each metabolite, which are separated from other metabolites according to (accurate) molecular mass. The fingerprint can be used as a classification tool. Some mass analyzers (e.g., fourier transform ion cyclotron resonance instruments, FT-ICR-MS) are capable of ultra-high resolution and permit the mass to be determined to four or five decimal places (Brown *et al.*, 2005). This allows empirical formulae to be assigned to peaks. Additionally, the coupling of high sensitivity with high resolution provides a rapid method of estimating the number of metabolites present and a valuable first indication, from the formulae, of their possible identities. Its main weakness is the inability to separate isomers of the same molecular mass (Pitt *et al.*, 2002).

***FT-ICR mass spectrometry.*** Fourier transform (FT)-ion cyclotron resonance (ICR) mass spectrometry (MS) has traditionally not been widely used. FT-ICR spectrum can measure many mass spectra per second, making it increasingly attractive in the pharmaceutical industry because of its ability to deliver more information per measurement (Brown *et al.*, 2005). FT-ICR provides ultra-high mass resolution and mass accuracy, non-destructive detection, high sensitivity and multi-stage MSn (Brown *et al.*, 2005). It has undergone rapid development and is now applied in many fields. High mass accuracy ensures rapid protein identification with high confidence based on single peptide mass measurements (Brown *et al.*, 2005). Many metabolites are in a mass range where FT-ICR can give immediate elemental composition, enabling direct identification without MS/MS, by comparison with public or locally generated databases (Zhang *et al.*, 2005).

***Multivariate statistical analysis (pattern recognition methods).*** Pattern recognition (PR) and related multivariate statistical approaches can be used to discern significant patterns in complex multivariate data sets and are particularly appropriate in situations where there are more variables than samples in the data set (Lindon *et al.*, 2004). The general aim of PR is to classify objects or to predict the origin of objects based on identification of inherent patterns in a set of measurements (Dunn *et al.,* 2005). PR methods can reduce the dimensionality of complex data sets via 2 dimensions (2D) or 3 dimensions (3D) mapping procedures, thereby facilitating the visualization of inherent patterns in the data. Methods such as principal components analysis (PCA) are termed 'unsupervised' techniques, in that no *a priori* knowledge of the class of the samples is required, and they are based on the calculation of latent variables (Weckwerth and Morgenthal, 2005). Principal components are linear combinations of the original descriptors, such that they are uncorrelated, and describe decreasing amounts of data variance (that is, PC1 > PC2 > PC3 and so on). Use of PCA enables the 'best' representation, in terms of biochemical variation in the data set, to be shown in 2D or 3D. In addition, multiparametric data can be modeled, so that the class of a sample from an independent data set can be predicted on the basis of a series of mathematical models that are derived from the original data or 'training' set. These methods are known as 'supervised' methods, and use class information to maximize the separation between classes (Weckwerth and Morgenthal, 2005). Supervised methods, such as soft independent modeling of classification analogy (SIMCA), partial least squares (PLS) analysis and PLS discriminant analysis (PLS-DA), can be used to predict objects that are unknown to the system on the basis of their NMR spectral properties or MS profiles, and are therefore valuable for generating models for predicting chemical toxicity, drug efficacy, disease status, and so on (Kim *et al.*, 2008; Quinones and Kaddurah-Daouk, 2009; Um *et al.*, 2009).

***Artificial neural networks (ANN).*** Artificial neural (or neuronal) networks (ANN) are simplified mathematical models of a biological neuronal system (Mao *et al.*, 1995). They 'learn' from existing data sets and are 'optimized' by specific algorithms (Mao *et al.*, 1995). Neurons can 'activate' their neighbors according to certain mathematical rules such as those developed by Hopfield (1982). They may then recognize familiar patterns, correct errors and remember sequences of events. An ANN takes the input data (e.g., gene expression data) and builds a network to predict either the categorical or continuous responses (Ripley *et al.*, 1996). They are 'robust' to a moderate amount of 'noise' in the data, but if the number of input variables (genes) > 1000, then the technique requires intensive computer-aid.

***Linear discriminant analysis (LDA).*** LDA is a statistical technique that can be used for the classification of individuals into mutually exclusive and exhaustive groups based on a set of independent variables. The LDA involves finding a linear combination of the independent variables that minimizes the probability of misclassifying the individuals into their respective groups (Goodacre, 2005).

## TOXICOLOGICAL APPLICATIONS

Using metabolomic technology, researchers are able to systematically determine metabolite concentration in a sample. This new technology has the potential for application in the areas of drug discovery/development and preventive screening/diagnostics (Table 4). Research continues to refine this technology in an effort to put these applications to use as quickly as possible (Lindon *et al.*, 2004). Metabolic profiling (of biological samples such as urine or blood plasma) can be generally used to determine the physiological changes induced by toxic effect of a chemical (or mixture of chemicals) (Kim *et al.*, 2008, 2009). The observed metabolic changes can be closely related to specific toxicity, e.g. specific hepatic or renal lesions (Nicholson *et al.*, 1985; Kim *et al.*, 2008; Park *et al.*, 2009). This is of particular interest to pharmaceutical companies that want to evaluate the toxicity of new drug candidates. If a new compound can be screened for adverse toxicity before it reaches clinical trials, then companies gain the advantage of being able to save the enormous cost of these trials (Lindon *et al.*, 2004). Metabolomics is emerging as an

**Table 4.** Application of metabolomics

| Application | | Ref. |
|---|---|---|
| **Drug Discovery** | - The major aim is to increase compound attrition in drug discovery.<br>- Reduce compound attrition in development and clinical analysis.<br>- Help explain reasons for toxicity.<br>- Produce expert system to identify toxicity. | Zhang *et al.*, 2005<br>Wang *et al.*, 2009 |
| **Biomarker identification** | - Markers of drug toxicity.<br>- Markers of drug efficacy. | Kim *et al.*, 2008<br>Nicholson *et al.*, 1985<br>Park *et al.*, 2009 |
| **Human disease diagnosis and personalized medicine** | | Yang *et al.*, 2004a, b<br>Clayton *et al.*, 2006 |
| **Agriculture** | - Measurement and identification of unintended effects of genetic modification. | Roessner *et al.*, 2001 |
| **Nutrition** | - Naturally functional foods. | Gidley *et al.*, 2004 |

excellent tool for elucidating the phenotype induced by a genetic modification, such as gene insertion or deletion. Metabolomics has the unique ability to relate direct phenotypic change with metabolic profile rather than genomics or proteomics (Holmes and Antti, 2002). It is more exciting to predict the function of unknown genes by comparing the metabolic perturbations induced by insertion/deletion of known genes. *Saccharomyces cerevisiae* and *Arabidopsis thaliana* are good model organisms for such advances in metabolomics and functional genomics (Allen *et al.*, 2003; Saito and Matsuda, 2008).

***Biomaker discovery for candidate drugs.*** One of the most interesting goals of pharmaceutical industry is to select robust new drug candidates for development or screen them for adverse effects in order to save money and time. Early preclinical screening of candidate drug toxicity is cost-effective and can suggest expected harmful side effects in clinical trials in drug development (Lindon *et al.*, 2004). Therefore, an important issue has been emerging to assess and screen toxicity of new molecular entities in early stages of new drug development. Biomarker discovery has been one of representative applications using metabolomics (Table 5). Consortium for Metabonomic Toxicology (COMET) has recently explored the evaluation of xenobiotic toxicity by biomarkers using metabolomics (Lindon *et al.*, 2003). COMET was formed between Imperial College London, UK and 6 major pharmaceutical companies to apply metabolomics data produced using $^{1}$H NMR spectroscopy of urine and blood serum samples to preclinical toxicological screening of candidate drugs (Ebbels *et al.*, 2007). Kim *et al.* (2008) reported that endogenous metabolites of allantoin, citrate, taurine, 2-oxoglutarate, acetate, lactate, phenylacetyl glycine, succinate, phenylacetate, 1-methylnicotinamide, hippurate, and benzoate were closely related to hepatotoxicity caused by $CCl_4$, acetaminophen, and D-galactosamine using 600 M $^{1}$H NMR spectroscopy. These results suggested that these endogenous metabolites could be used as putative biomarkers for preclinical hepatotoxicity. Recently, high throughput toxicity and safety screening methods were developed through a combination of metabolite profiles (Ebbels *et al.*, 2007). Schoonen *et al.* (2007) reported that the total NMR dataset of rat urine showed more sensitivity to liver toxicity caused by bromobenzine and paracetamol than histopathology and clinical chemistry.

In addition, phospholipid metabolic profiles were studied and potential biomarkers were identified in rat plasma using HPLC-MS after $\gamma$-irradiation exposure (Wang *et al.*, 2009). Phosphatidylethanolamine and phosphatidylserine were assigned to be biomarkers for exposure to gamma-rays. Metabolomic investigation of toxicity caused by doxorubicin (adriamycin) was performed in rats using NMR spectroscopy (Park *et al.*, 2009). Presence of trimethylamine N-oxide (TMAO), glucose, lactate, alanine, and valine were related to renal toxicity induced by doxorubicin and creatine, phentlacetylglycine, N-methylnicotinic acid, and hippurate levels were suggestive of liver injury (Park *et al.*, 2009). Metabolic profiling of realgar in rats was recently studied for toxicological effects using NMR spectroscopy (Wei *et al.*, 2009). Various endogenous metabolites were suggested as biomarkers correlated to liver and kidney toxicity caused by realgar. Metabolomic studies for

**Table 5.** Metabolomic biomarkers associated with target organ toxicity

| Target organ | Toxicants | Biomarkers | Ref. |
|---|---|---|---|
| Liver toxicity | Allyl alcohol | ↑ creatinine, lactate, phenylacetyl glycine,<br>↓ N-methyl nicotinamide, taurine | Beckwith-Hall *et al.*, 1998 |
| | Bromobenzene | ↑ 5-oxoproline, glucose, acetate, lactate<br>↓ citrate, $\alpha$-ketoglutarate, succinate | Waters *et al.*, 2006 |
| | $\alpha$-Naphthylisocyanate | ↑ taurine, creatine, glucose<br>↓ citrate, $\alpha$-ketoglutarate, succinate | Waters *et al.*, 2001 |
| | Methapyrilene | ↑ succinate, triglyceride, dimethylglycine, trimethylamine-N-oxide<br>↓ glucose, glycogen | Craig *et al.*, 2006 |
| | Hydrazine | ↑ $\beta$-alanine, 3-D-hydroxybutyrate, citrulline, N-acetyl-citrulline<br>↓ trimethylamine-N-oxide | Bollard *et al.*, 2005 |
| Renal toxicity | Gentamicin | ↑ glucose<br>↓ trimethylamine-N-oxide | Lenz *et al.*, 2005 |
| | Cisplatin | ↑ alanine, leucine, glucose, 2-oxoglutarate, pyruvate, valine<br>↓ trimethylamine | Garrod *et al.*, 2001 |
| | Mercuric chloride | ↑ acetate, amino acids, glucose, organic acids<br>↓ citate, creatinine, hippurate, $\alpha$-ketoglutarate, succinate. | Nicholson *et al.*, 1985 |

investigation of biomarker discovery have been carried out and several endogenous metabolites were useful for prediction or screening of specific toxic effects caused by chemicals.

***Connections to genomics and proteomics.*** Metabolomics can be very useful for functional genomics and proteomics. Many companies that invested in genomic and proteomic approaches have augmented their work with metabolomic technologies (Nicholson *et al.*, 1999). For example, if a company is interested in a specific gene, but it is not sure what the gene product does, it can create a gene knock-out and compare its metabolic profile against that of the wild-type. This will give direct information as to the function of a protein and its coding gene. There is a linear progression of biological events, from genomics to metabolomics, which opens a new post-genomic era and a new approach to elucidate mechanisms of toxicities in toxicological research: e.g. the analysis of genetic variation (toxicogenomics), gene expression (transcriptomics), gene products (toxicoproteomics) and their metabolic effects (metabolomics) (Kell, 2004). The genetic approach to unravel the complexity inherent within human biology is very limited. But, it provides only one layer of complexity. To understand common human disease traits, the integration of multiple 'omics' datasets is required (Zhu *et al.*, 2007). As seen in recent literature, there have been attempts to combine two datasets related to clinical phenotype, such as pharmacogenomics, toxicogenomics, epigenetics, and metagenomics. Recently, combining genetics and metabolomics has been applied. Cholesterol-associated susceptible genes for Alzheimer's disease were identified by combining genetics with sterol metabolic profiling (Papassotiropoulos *et al.*, 2005). The ratio of metabolites was used to identify the function of putative genes (Gieger *et al.*, 2008). Stylianou and colleagues (2008) linked proteomics to quantitative trait loci (QTL) to identify changes in function rather than the protein quantification. To elucidate complex biological phenomenon, combining multiple types of technologies, including genetics, transcriptomics, proteomics and metabolomics, should be performed.

***Disease diagnosis.*** Recently, metabolomics studies have been used to screen the patients for general metabolic disorders using NMR analysis (van Doorn *et al.*, 2007). There has been a report that analysis of serum lipid profiles using NMR could discriminate tumors (83%) and non-tumors (8%) from 52 patients diagnosed as coronary heart disease (Brindle *et al.*, 2002). Colon cancer and coronary heart disease could be precisely diagnosed from healthy volunteers through neural network analysis and classification of NMR spectra of lipoprotein fractions (Bathen *et al.*, 2000). Moreover, testosterone and its metabolites, epitestosterone and dihydrotestosterone were measured from human hair using GC-MS (Choi *et al.*, 2001). Hair samples from bald men presented separate metabolic profiles from healthy volunteers and testosterone/epitestosterone ratio was suggested as biochemical biomarker useful for diagnosis of early baldness (Choi *et al.*, 2001). Research on the correlation between cervical cancer and urinary polyamines and endogenous steroids using MS, showed that the ratios of 16$\alpha$-hydroxyestrone/2-hydroxyestrone, 5$\beta$-tetrahydrocortisol/5a-tetrahydrocortisol, and putresine/N-acetylspermidine were very crucial for diagnosis of cervical cancer (Lee *et al.*, 2003). After liver fibrosis and cirrhosis were progressively induced by thioacetamide in rats, liver extracts and serum were analyzed using $^1$H NMR to characterize the stage of fibrosis (Constantinou *et al.*, 2007). PCA profiles from lipid liver extract and serum showed the progress of fibrosis and cirrhosis. Serum leucine, isoleucine, valine, lactate, alanine, acetate, acetoacetate, glutamine, trimethylamine, creatine, and glucose were sensitively influenced during liver fibrosis and cirrhosis caused by thioacetamide and this study could be extended in clinical diagnosis for management of cirrhotic patients (Constantinou *et al.*, 2007). Cerebrospinal fluid (CSF), a clear bodily fluid that occupies the subarachnoid space and the ventricular system around and inside the brain, can serve as a metabolomic sample for investigating Alzheimer's disease, meningitis, and so on. CSF samples were used to distinguish normal subjects from those with meningitis using NMR spectroscopy (Coen *et al.*, 2005). Early detection of oral cancer using NMR spectroscopy was studied in patients' serum (Tiziani *et al.*, 2009). Serum metabolite profile completely discriminated cancer patients from control group and also between different stages of oral cancer (Tiziani *et al.*, 2009). Normal colon tissues and colorectal cancer tissues were analyzed with GC-TOF (time-of-flight)-MS to investigate metabolomic profiling of human colon carcinoma (Denkert *et al.*, 2008). In cancer, intermediates of the TCA cycle and lipids were down-regulated, whereas urea cycle metabolites, purines, pyrimidines and amino acids were up-regulated compared to normal samples (Denkert *et al.*, 2008).

***Risk assessment.*** Metabolomics can be of great interest to risk assessment of toxic substances. There are two advantages of metabolomics for risk assessment: understanding molecular mechanisms of toxicity and sensitive exposure assessment. Kim *et al*. (2009) studied the exposure assessment of methoxyclor, an organochlorine pesticide, to rats using urinary NMR spectral data and compared it with traditional exposure of methoxyclor, using uterotrophic assay, in ovariectomized female rats for 3 consecutive days. Metabolomics dataset was much more sensitive to methoxyclor rather than traditional uterotrophic assay and the endogenous metabolites of acetate, alanine, benzoate, lactate and glycine were suggested as putative exposure biomarkers for an endocrine disruptor of methoxyclor (Kim *et al*., 2009). Nonylphenol is an environmental contaminant and endocrine disrupting chemical. Lee *et al*. (2007) investigated the metabolomic profiles for endocrine toxic effects caused by nonylphenol, using GC-MS. Urinary tetrahydrocorticosterone and 5$\alpha$-tetrahydrocorticosterone were suggested as possible biomarkers of nonylphenol-induced toxicity or exposure.

***Future prospects and concluding remarks.*** Metabolomics has been considered an emerging and promising technology in toxicological research, although its validation is required. Using metabolomics, the data has shown that combinational biomarkers for toxicity or disease were identified, which were able to monitor the toxicity or efficacy of chemicals or drugs in preclinical or clinical trials. Also, new biochemical assays for disease diagnosis were derived using NMR or MS analysis. As one part of multiple 'omics' technologies, metabolomics plays an important role in understanding biological phenomenon because it's very close to the phenotype of biological effects. Risk assessment is a very promising area to explore toxic mechanism or exposure assessment using metabolomics. One thing that needs to be pointed out for metabolomics is totally based on analytical method and multivariate analysis. Therefore, the analytical procedures should be stable, robust, and highly reproducible. Multivariate analysis can turn complex dataset into readable and interpretable. In contrast to other 'omics', metabolomics has the advantage of having a good level of biological reproducibility, low cost of per sample, minimal invasion of sampling, and direct identification of phenotypes with real biological endpoints. However, it requires a further research for validation, specificity, and sensitivity for the prediction of toxic manifestations. In addition, a comparative relationship between conventional biomarkers and new biomarkers derived from metabolomics should be needed.
